# Assessing financial toxicity with COST-FACIT: experience from a tertiary care cancer center

**DOI:** 10.1007/s00520-026-11344-z

**Published:** 2026-10-17

**Authors:** Mahir Khan, Elizabeth N. Dewey, Colleen Strzalka, Aarushi Bhan, Heather McKee Hurwitz, Holly Stegh, Renata Hurn, Jacqueline Tomer, Sujata Patil, Kim Bell, Betty K. Hamilton

**Affiliations:** 1 Advocate Health Care, Oak Lawn, IL USA; 2 Center for Populations Health Research, Quantitative Health Sciences, Cleveland Clinic, Cleveland, OH USA; 3 Department of Hematology and Oncology, Taussig Cancer Institute, Cleveland Clinic, Cleveland, OH USA; 4 Case Western University, Cleveland, OH USA; 5 Quantitative Health Sciences, Cleveland Clinic, Cleveland, OH USA; 6 Blood and Marrow Transplant Program, Department of Hematology and Oncology, Taussig Cancer Institute, Cleveland Clinic, 9500 Euclid Ave CA60, Cleveland, OH 44195 USA

**Keywords:** Cancer, Financial toxicity, Quality of life

## Abstract

**Purpose:**

Financial toxicity (FT) refers to the direct and indirect consequences of cancer diagnosis and treatment and is a key mediator of quality of life (QOL) and survival in cancer patients. To address this, financial navigation (FN) programs have been implemented in many cancer centers. At our large tertiary care cancer center, the FN program uses the Comprehensive Score for Financial Toxicity (COST) to screen patients undergoing first cancer treatment. We aimed to better understand FT, risk factors, and association with outcomes in our cancer population.

**Methods:**

We identified 1906 patients with any cancer diagnosis and a completed COST between 6/2021 and 2/2024. Sociodemographic data including the Area Deprivation Index (ADI), disease, and treatment characteristics as well as patient-reported outcomes (PROMIS10, PHQ9 and NCCN DT) were collected. Risk factors for FT and association of FT on overall survival (OS) were analyzed.

**Results:**

Median age was 65 years (IQR 56–72); 54% female, 82% white, 57% married, 47% insured privately, and 35% with Medicare. Younger age, race/ethnicity, insurance type, tobacco use, and higher ADI were associated with higher FT. Higher distress (NCCN DT, *N* = 1001) and lower physical (*N* = 1474) and mental (*N* = 1505) health (PROMIS10) were also associated with higher FT. FT was not associated with time to treat (TTT) or OS. However, higher ADI was significantly associated with TTT (HR 0.98, 95% CI 0.96–1.00, *p* = 0.04) but not OS.

**Conclusions:**

Our study provides insights into risk factors for FT in a large cancer population. We did not demonstrate a significant association of FT on survival. Further analysis in specific disease groups is needed to better understand these relationships.

**Supplementary Information:**

The online version contains supplementary material available at https://doi.org/10.1007/s00520-026-11344-z.

## Introduction

The cost of cancer care in the USA has significantly increased over the last several decades for patients and their families. With the rapid advancement of novel diagnostics and therapeutics, the costs of treatment continue to rise [1, 2]. Financial toxicity (FT) is a term referring to a patient’s direct treatment expenses, loss of income due to job loss and unexpected leave, and indirect costs associated with completing treatment like childcare, travel to the facility, and meals while onsite [3–5]. FT is associated with poor quality of life, symptom burden, barriers to adherence to medications, and survival [6, 7]. Given the increasing awareness of the risk of financial burden in cancer patients, the financial concerns of patients, caregivers, physicians, health systems, and payers are increasingly being investigated [4]. Thus, research in defining and measuring the consequences of cancer care has increased significantly in recent years.

Several studies have revealed cancer patients’ experiences with financial toxicity. Cancer patients experience financial debt at a significantly higher rate than the general population and are more likely to file for bankruptcy, experience foreclosures, collections, and repossessions [8, 9]. Patients with lower household income, racial/ethnic minority status, live in a rural location, or are uninsured are at an increased risk of FT. Furthermore, patients who experience high FT subsequently have a higher likelihood of medication non-adherence, delayed medical care [10–12], and ultimately, higher mortality [1, 13]. FT has also been shown to be associated with health-related quality of life (HRQOL) including increased anxiety, depression, and symptom burden [9, 14–16].

Financial navigation (FN) programs have emerged as a strategy to recognize and mitigate FT. These programs address financial toxicity at multiple levels, offering services such as case management, transportation support, financial education, and assistance with insurance and grant applications [17]. Within our cancer institute, we have an established FN team consisting of a patient financial advocate, a financial navigator, and social worker, who collaboratively assess financial and related psychosocial needs to address barriers and access to care. Since 2021, the FN team has been using the Comprehensive Score for Financial Toxicity- Functional Assessment of Chronic Illness Therapy (COST-FACIT) [18] to identify patients who are planning to receive cancer therapy and may benefit from additional resources such as grants, pharmaceutical programs, and co-pay assistance for medications. To better understand the burden of FT within our cancer population, we undertook this study to describe FT, risk factors for higher FT, and association with HRQOL in a large cohort of cancer patients.

## Methods

We conducted a retrospective study of patients who were seen for a cancer diagnosis and completed a COST-FACIT survey at the Cleveland Clinic Cancer Institute from June 2021 to February 2024. The primary objectives of the study were to describe FT and severity of FT in our cancer population, and identify sociodemographic, disease, and treatment-related risk factors for FT. Secondary objectives were to analyze association of FT on time to treat and overall mortality. FT and additional patient-reported outcome measures (PROM), as well as patient demographic, disease, and treatment data were obtained from the institutional Tumor Registry Database and Electronic Health Record (EHR). The study was approved by the Cleveland Clinic Institutional Review Board.

The COST-FACIT is a validated and standardized PROM that evaluates FT using an 11-item questionnaire. The score ranges from 0 to 44 with lower scores indicating higher financial toxicity. The score was categorized into 4 grades based on previous validation on impact on quality of life: grade 0, no impact (COST-FACIT ≥ 26); grade 1, mild impact (COST-FACIT 14–25); grade 2, moderate impact (COST-FACIT 1-13); and grade 3, high impact (COST-FACIT 0) [19]. The COST-FACIT was offered to patients at the time of first outpatient treatment for cancer diagnosis. Only initial COST-FACIT data was used for analysis, as there were limited follow-up surveys. Additional PROM data were collected closest to the date of the COST-FACIT based on the last appointment where vital signs were recorded. PROMs are routinely collected at the cancer center from a patient’s initial visit and subsequently on 84-day intervals. They include the Patient Reported Outcomes Measurement Information System (PROMIS)−10, the National Comprehensive Cancer Network Distress Thermometer (NCCN DT), and the Patient Health Questionnaire (PHQ)−9. The PROMIS-10 is a 10-item questionnaire designed to assess a patient’s overall health status. It includes questions to measure physical and mental health status. Higher scores typically indicate better physical and mental health (> 58 excellent; 50–58 very good; 42–9 good; 35–41 fair; < 35 poor). NCCN DT is a validated single-item question tool for initial screening of distress with a rating scale from 0 to 10. Higher scores indicate higher distress (0–3 mild; 4–7 moderate; 8–10 severe). The PHQ-9 is a nine-item questionnaire which screens for depression. It consists of an initial 2 questions (PHQ-2), and if screening criteria are met for depression, it expands to 9 questions (PHQ-9). Higher scores also indicate more severe depression (0–4 minimal; 5–9 mild; 10–14 moderate; 15–19 moderate-severe; 20–27 severe).

Sociodemographic variables of interest included: age, sex, race/ethnicity, marital status, smoking history, body mass index (BMI), insurance information, and area deprivation index (ADI). The ADI is a validated index of neighborhood deprivation and disadvantage taking into account factors related to income, education, employment, and housing quality [20]. The ADI was derived from 2013–2017 five-year American Community Survey (ACS) estimates [21]. For analysis, ADI was grouped into quintiles based on the percentile rank in Ohio for descriptive summaries. The higher the ADI, the higher the socioeconomic disadvantage in the neighborhood/community. Although neighborhood deprivation measured by ADI is associated with socioeconomic disadvantage, it reflects community-level characteristics rather than an individual patient's perceived financial burden. We therefore evaluated ADI alongside COST-FACIT to determine whether neighborhood disadvantage and patient-reported financial toxicity demonstrated independent associations with clinical outcomes. Regression models treated ADI percentile rank as a continuous variable to maximize statistical power and estimate changes in risk per 5-percentile (Table 2) or 10-percentile (Table 3) increase. Data on patient’s disease, staging, and treatment were based on the date of diagnosis. Missing data were not imputed. PROMs were not considered appropriate for imputation because they represent individualized patient-reported outcomes. Analyses were therefore conducted using available-case data. Levels with sparse observations were combined, assigned to an “Unknown” category, or excluded when appropriate.

### Statistical analysis

Standard descriptive statistics were used to describe sociodemographic, disease, and treatment factors by financial toxicity groups. Continuous factors are summarized as medians and interquartile ranges, and comparisons between groups were evaluated with log-transformed linear regression. Categorical factors are summarized using counts and percentages, and comparisons between availability responses were evaluated using Kruskal–Wallis test, Chi-Square tests, and Fisher’s Exact test, as appropriate.

Missing data was not imputed. Most of the missing data was in PROMs and was not considered suitable for imputation due to the individualistic nature of the scores. Additionally, levels in some categorical variables were combined or excluded from hypothesis testing due to low sample size or missingness.

Financial toxicity was modeled as an ordinal outcome using the four validated COST-FACIT categories (no, mild, moderate, and high financial toxicity), with cumulative logistic regression estimating the odds of belonging to a higher financial toxicity category. Odds ratios represent the odds of being in a higher financial toxicity category. Variables with univariable *p *< 0.20 were considered for multivariable modeling using the variable selection approach described by Hosmer and Lemeshow [22]. All odds ratios were adjusted for diagnosis.

Overall patient survival was evaluated for all patients and stratified by tumor type (hematologic malignancy or solid tumor) using Cox Proportional Hazards adjusting for patient characteristics age, BMI, sex, race/ethnicity, primary payer, marital status, smoking status, time to first treatment (from date of diagnosis), clinical stage (categorized as stage 0–I, II, III, IV, or Unknown), and chemotherapy treatment. The proportional hazards assumption was verified using Schoenfeld residuals.

A sensitivity analysis was performed to examine the demographic differences between populations of patients who did and did not fill out a COST-FACIT survey or a PROM. Cohorts were compared using standard descriptive statistics, and differences were evaluated with Kruskal–Wallis test, Chi-Square tests, and Fisher’s Exact test, as appropriate. Because analyses were exploratory and based on prespecified clinical hypotheses, no formal adjustment for multiple comparisons was performed. All analysis was done using SAS 9.4 (SAS Institute, Cary, NC). Significance was assessed at *p* < 0.05.

## Results

### Patient and disease characteristics

We identified 16,814 unique patients seen for at least one appointment between June 2021 and February 2024. Of these, 1906 had completed at least 1 COST-FACIT assessment and met inclusion criteria (Supplementary Fig. 1).

Patient sociodemographics including PROMs are shown in Table 1. Median age of the cohort was 65 years (IQR 56–72) and *N* = 1033 (54.2%) were female. The majority of the cohort was non-Hispanic White, *N* = 1561 (81.9%). Over half of the patients reported being married, *N* = 1091 (57.2%) and *N* = 896 (47%) had private insurance. The most common malignancies were gastrointestinal (*n* = 347, 18.2%), breast (*n* = 305, 16%), lymphoma (*n* = 245, 12.9%), lung (*n* = 230, 12.1%), and myeloma (*n* = 196, 10.3%). The median time from diagnosis to COST-FACIT survey was 88 days, range 46 to 303; with the larger range reflective of the heterogeneity of diseases and treatment timing; and inclusion of malignancies in which treatment may have begun in the inpatient setting. Additional disease and treatment characteristics are detailed in Supplementary Table 1.

**Table 1:** Demographic characteristics and patient-reported outcomes of patients with COST survey

Factor	Overall (*N* = 1,906)		No impact (*N* = 815)		Mild impact (*N* = 632)		Moderate impact (*N* = 414)		High impact (*N* = 45)		*p*-value
	***N***	***N* (%)**	***N***	***N* (%)**	***N***	***N* (%)**	***N***	***N* (%)**	***N***	***N* (%)**	
Age (median, IQR)	1906	65.0 [56.0, 72.0]	815	68.0 [60.0, 74.0]	632	64.0 [56.0, 71.0]	414	60.0 [50.0, 68.0]	45	62.0 [52.0, 67.0]	** < *0.001***^***b***^
Sex	1905		815		632		413		45		***0.030***^***c***^
FEMALE		1033 (54.2)		418 (51.3)		342 (54.1)		249 (60.3)		24 (53.3)	
MALE		872 (45.8)		397 (48.7)		290 (45.9)		164 (39.7)		21 (46.7)	
Race/Ethnicity	1906		815		632		414		45		** < *0.001***^***c***^
Non-Hispanic White		1561 (81.9)		725 (89.0)		502 (79.4)		307 (74.2)		27 (60.0)	
Non-Hispanic Black		237 (12.4)		54 (6.6)		91 (14.4)		78 (18.8)		14 (31.1)	
Non-Hispanic Asian		26 (1.4)		12 (1.5)		8 (1.3)		5 (1.2)		1 (2.2)	
Non-Hispanic Other		8 (0.42)		0 (0.00)		4 (0.63)		4 (0.97)		0 (0.00)	
Hispanic		45 (2.4)		8 (0.98)		17 (2.7)		17 (4.1)		3 (6.7)	
Unknown/Declined		29 (1.5)		16 (2.0)		10 (1.6)		3 (0.72)		0 (0.00)	
Marital status	1906		815		632		414		45		** < *0.001***^***c***^
Single		406 (21.3)		107 (13.1)		150 (23.7)		132 (31.9)		17 (37.8)	
Married		1091 (57.2)		537 (65.9)		339 (53.6)		194 (46.9)		21 (46.7)	
Divorced/Separated/Widowed		388 (20.4)		161 (19.8)		134 (21.2)		86 (20.8)		7 (15.6)	
Other/Unknown		21 (1.1)		10 (1.2)		9 (1.4)		2 (0.48)		0 (0.00)	
Primary payer	1906		815		632		414		45		** < *0.001***^***c***^
Medicare		665 (34.9)		283 (34.7)		230 (36.4)		139 (33.6)		13 (28.9)	
Medicaid		211 (11.1)		33 (4.0)		85 (13.4)		81 (19.6)		12 (26.7)	
Private		896 (47.0)		451 (55.3)		274 (43.4)		154 (37.2)		17 (37.8)	
Tricare		22 (1.2)		15 (1.8)		4 (0.63)		3 (0.72)		0 (0.00)	
Other		25 (1.3)		8 (0.98)		7 (1.1)		8 (1.9)		2 (4.4)	
Unknown		87 (4.6)		25 (3.1)		32 (5.1)		29 (7.0)		1 (2.2)	
Smoking history	1906		815		632		414		45		** < *0.001***^***c***^
Current		263 (13.8)		87 (10.7)		93 (14.7)		74 (17.9)		9 (20.0)	
Former		808 (42.4)		329 (40.4)		279 (44.1)		181 (43.7)		19 (42.2)	
Never		817 (42.9)		393 (48.2)		255 (40.3)		152 (36.7)		17 (37.8)	
Unknown		18 (0.94)		6 (0.74)		5 (0.79)		7 (1.7)		0 (0.00)	
BMI	1905	26.9 [23.4, 30.5]	814	26.8 [23.5, 30.6]	632	26.9 [23.6, 30.3]	414	27.1 [23.2, 32.8]	45	26.7 [21.3, 30.3]	0.65^b^
Deceased	1906	377 (19.8)	815	155 (19.0)	632	132 (20.9)	414	80 (19.3)	45	10 (22.2)	0.80^c^
Recurrence	1906	96 (5.0)	815	45 (5.5)	632	29 (4.6)	414	21 (5.1)	45	1 (2.2)	0.70^c^
*Patient Report Outcome Measures (PROMs)*											
Cost Facit Score	1906	23.0 [14.0, 32.0]	815	34.0 [29.0, 38.0]	632	20.0 [16.8, 22.0]	414	8.0 [5.0, 10.0]	45	0.00 [0.00, 0.00]	** < *0.001***^***b***^
Days from diagnosis to Cost Facity survey	1906	88.0 [46.0, 303.0]	815	84.0 [46.0, 238.0]	632	85.0 [46.0, 295.0]	414	115.5 [50.0, 449.0]	45	58.0 [36.0, 514.0]	***0.013***^***b***^
NCCN Score	1001	3.0 [0.00, 5.0]	446	2.0 [0.00, 4.0]	324	3.0 [0.00, 6.0]	212	4.0 [0.00, 6.0]	19	5.0 [3.0, 7.0]	** < *0.001***^***b***^
PHQ 9 Score	1416		641		459		287		29		** < *0.001***^***c***^
0		849 (60.0)		424 (66.1)		261 (56.9)		153 (53.3)		11 (37.9)	
1		204 (14.4)		79 (12.3)		65 (14.2)		53 (18.5)		7 (24.1)	
2		271 (19.1)		109 (17.0)		95 (20.7)		57 (19.9)		10 (34.5)	
3		32 (2.3)		12 (1.9)		15 (3.3)		4 (1.4)		1 (3.4)	
4		31 (2.2)		9 (1.4)		10 (2.2)		12 (4.2)		0 (0.00)	
5		7 (0.49)		2 (0.31)		4 (0.87)		1 (0.35)		0 (0.00)	
6		22 (1.6)		6 (0.94)		9 (2.0)		7 (2.4)		0 (0.00)	
PROMIS-10 MENTAL	1474	45.8 [41.1, 53.3]	656	48.3 [43.5, 53.3]	468	45.8 [41.1, 50.8]	315	43.5 [38.8, 48.3]	35	43.5 [36.3, 48.3]	** < *0.001***^***b***^
PROMIS-10 PHYSICAL	1505	44.9 [37.4, 50.8]	672	47.7 [39.8, 54.1]	480	44.9 [37.4, 50.8]	317	42.3 [34.9, 47.7]	36	37.4 [36.2, 44.9]	** < *0.001***^***b***^

Statistics presented as median [P25, P75], *N* (column %)

*p*-values: *b* = Kruskal–Wallis test, *c* = Pearson's chi-square test

### Financial toxicity

**Fig. 1:**
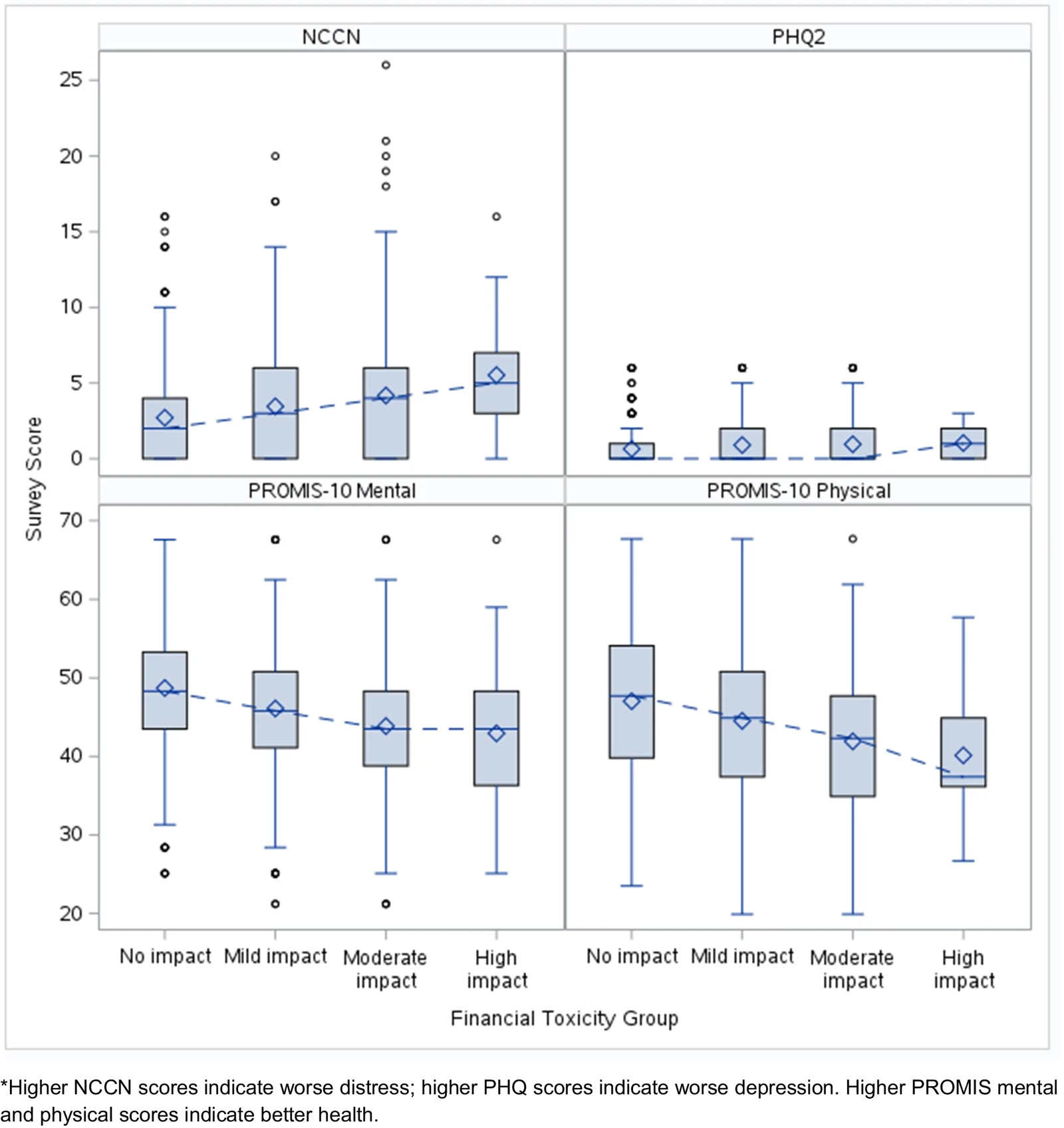
Financial toxicity and patient-reported outcomes

Of 1906 patients, *N* = 815 (43%) reported no FT at initial treatment, while *N* = 632 (33%) reported low, *N* = 414 (22%), moderate, and *N* = 45 (2%) high FT. Several baseline characteristics were associated with higher FT categories: younger age (*p* < 0.001), female sex (*p* = 0.03), Black race (*p* < 0.001), Hispanic ethnicity (*p* < 0.001), single marital status (*p* < 0.001), having Medicaid insurance (*p* < 0.001), smoking (*p* < 0.001), and higher ADI (*p* < 0.001). Of note, although Hispanic ethnicity was noted to be associated with higher FT, this is limited by very small numbers. PROMs were also associated with higher FT: higher distress as measured by NCCN DT (*p* < 0.001), depression as measured by higher PHQ-9 scores (*p* < 0.001), and lower HRQOL as measured by lower PROMIS-10 mental and physical scores (*p* < 0.001) (Fig. 1).

There was no significant association between FT and cancer diagnosis, although a relatively higher proportion of high-impact FT patients had breast (24.4%), lymphoma (22.2%), and myeloma (13.3%) diagnoses (Supplementary Table 1). Disease stage and treatment were not significantly associated with FT, except for those who received both chemotherapy and hormone therapy; there were fewer patients with no FT (11.9%) compared to mild FT (17.4%) (*p* = 0.01).

### Sensitivity analysis

To determine whether the cohort of patients with a COST-FACIT and/or PROM was representative of our cancer population, we performed sensitivity analyses evaluating differences between patients with a COST-FACIT survey and without (Supplementary Table 2). Patients with a completed COST-FACIT survey versus no COST-FACIT were younger (median age 65 versus 69 years, *p* < 0.001), had lower BMI (26.9 versus 27.4, *p* = 0.03), were more likely to be non-Hispanic White (82% versus 79%, *p* < 0.001), and have Medicare insurance (35% versus 13%, *p* < 0.001). Patients who completed a COST-FACIT survey also reported higher median NCCN DT score (3 versus 2, *p* = 0.03), and in general were more likely to complete other PROMs (Supplementary Table 2).

### Risk factors for increasing financial toxicity

Several factors were associated with greater financial toxicity at time of initial treatment (Table 2). In univariable analysis, younger age (*p* < 0.001), female sex (*p* = 0.02), non-Hispanic Black (NHB) and Hispanic race, and ethnicity compared to non-Hispanic White (*p* < 0.001), Medicaid and Medicare payer (*p* < 0.002), smoking (*p* < 0.001), single marital status (*p* < 0.001), lower physical/mental well-being (*p* < 0.001), and increased distress (*p* < 0.001) were all associated with increasing FT. In multivariable analysis, younger age (decreasing per 5 years), (OR 1.18, 95% CI 1.12–1.23, p < 0.001); NHB vs non-Hispanic White (NHW), (OR 1.62, 95% CI 1.19–2.20, *p* = 0.002); Hispanic vs NHW (OR 2.0, 95% CI 1.11–3.59, *p* = 0.02); Medicaid vs Private insurance (OR 2.35, 95% CI 1.68–3.29, *p* < 0.001); Medicare vs Private insurance (OR 1.85, 95% CI 1.47–2.33, *p* < 0.001); former or current history of smoking (OR 1.34, 95% CI 1.08–1.66, *p* = 0.007 and OR 1.41, 95CI 1.05–1.89, *p* = 0.02); and increasing ADI, per 5%-ranks, (OR 1.04, 95% CI 1.02–1.06, *p* < 0.001) all remained significantly associated with higher FT.

**Table 2:** Risk factors for increasing financial toxicity (ordinal cumulative logistic regression)

Comparison	Univariable				Multivariable			
	Odds ratio	Lower limit	Upper limit	*p*-value	Odds ratio	Lower limit	Upper limit	*p*-value
Age per 5-year decrease	1.17	1.13	1.21	** < *0.001***	1.18	1.12	1.23	** < *0.001***
Sex (F v M)	1.259	1.038	1.528	***0.02***	1.17	0.94	1.46	0.15
Race/ethnicity: NHB vs NHW	2.686	2.074	3.478	** < *0.001***	1.62	1.19	2.20	***0.002***
Race/ethnicity: Hispanic vs NHW	3.245	1.872	5.623	** < *0.001***	2.00	1.11	3.59	***0.02***
Race/ethnicity: other vs NHW	1.048	0.654	1.680	0.85	0.94	0.53	1.66	0.83
Payer: Medicaid vs private	3.997	3.009	5.310	** < *0.001***	2.35	1.68	3.29	** < *0.001***
Payer: Medicare vs private	1.355	1.121	1.638	***0.002***	1.85	1.47	2.33	** < *0.001***
Payer: other vs private	1.929	1.378	2.701	** < *0.001***	1.85	1.28	2.69	***0.001***
Smoking: former vs never	1.404	1.161	1.698	** < *0.001***	1.34	1.08	1.66	***0.007***
Smoking: current vs never	1.946	1.500	2.526	** < *0.001***	1.41	1.05	1.89	***0.02***
ADI (per 5%-ranks)	1.078	1.060	1.095	** < *0.001***	1.04	1.02	1.06	** < *0.001***
Smoking: former vs current	0.721	0.558	0.933	***0.01***	0.95	0.72	1.27	0.75
Single vs married	2.513	2.030	3.112	** < *0.001***	1.21	0.93	1.57	0.16
D/S/W vs married	1.327	1.068	1.648	***0.01***	1.21	0.94	1.55	0.14
Single vs D/S/W	1.894	1.463	2.453	** < *0.001***	1.00	0.74	1.36	0.99
PROMIS-10 Physical (per 5 points)	0.793	0.752	0.836	** < *0.001***	-	-	-	-
PROMIS-10 Mental (per 5 points)	0.790	0.747	0.835	** < *0.001***	-	-	-	-
NCCN (per 1 point)	1.111	1.074	1.150	** < *0.001***	-	-	-	-
Chemo: multiple agents vs single agent	0.991	0.765	1.283	0.94	-	-	-	-
Stage: IV vs III	1.105	0.788	1.550	0.56	-	-	-	-
Stage: III vs II	1.020	0.707	1.470	0.92	-	-	-	-
Stage: II vs 0/I	1.050	0.756	1.460	0.77	-	-	-	-
Chemo: multiple agents vs none	1.063	0.878	1.286	0.53	-	-	-	-
Chemo: single agent vs none	1.073	0.814	1.414	0.62	-	-	-	-
BMI (per 1)	1.003	0.991	1.015	0.64	-	-	-	-

Statistics presented as N (column %). *p*-values: c=Pearson's chi-square test

### Survival and time to first treatment following diagnosis

**Fig. 2:**
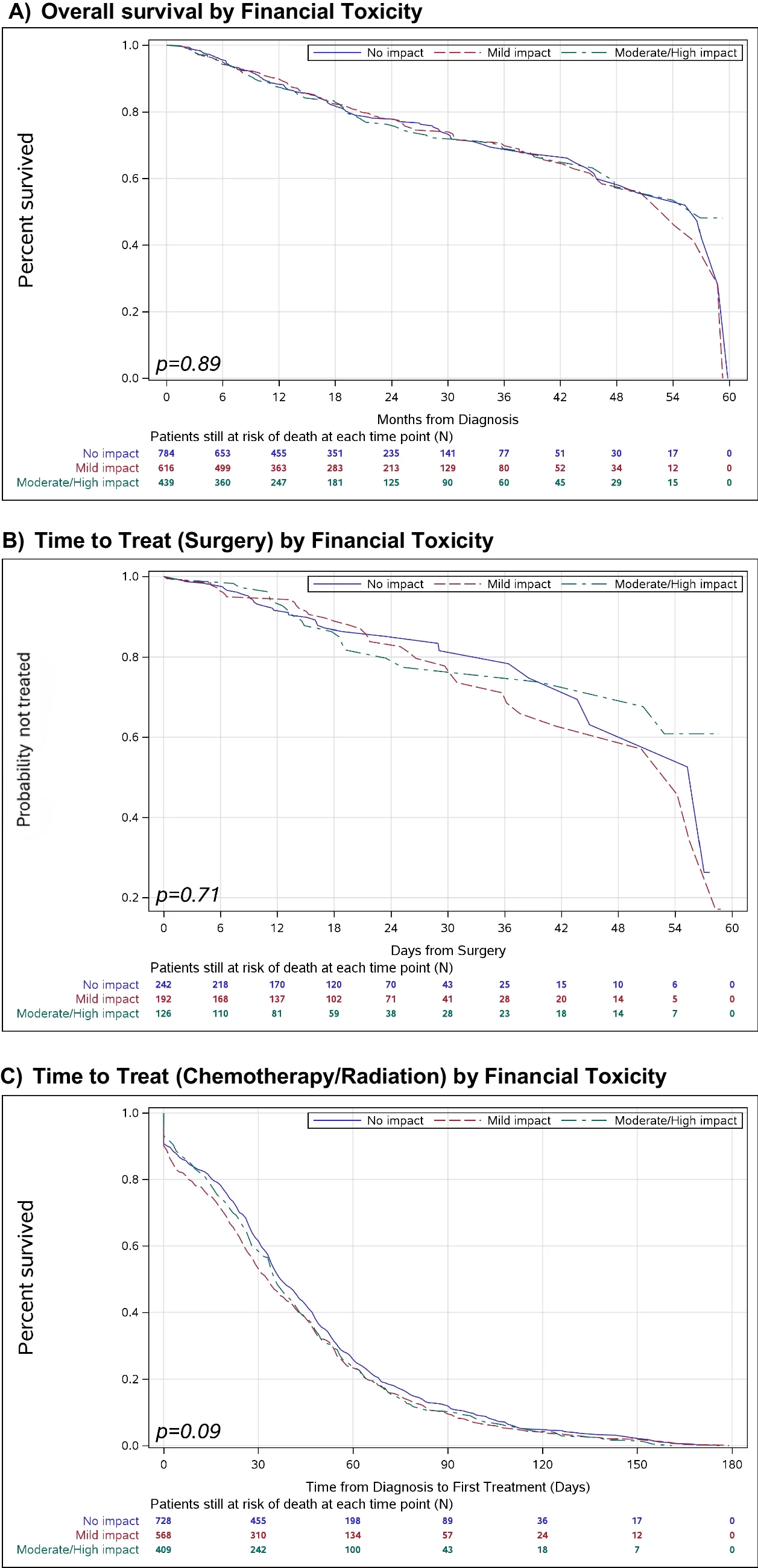
Survival and time-to-treat outcomes

Overall survival (OS) and time-to-treat outcomes among FT groups are shown in Fig. 2. In the entire cohort, we found no significant differences in overall survival among no, mild, and moderate/high FT (Fig. 2A). There was also no difference among FT groups and time-to-treat (Fig. 2B and C).

We further investigated risk factors for survival and time to treat, with FT as the main variable of interest. Given the association of FT and ADI, and previous findings that ADI was associated with cancer care outcomes, we included both variables of interest in the model. (Table 3). While there was no difference in OS among FT categories in the entire cohort in multivariable analysis, we found an increased risk of mortality in patients reporting moderate or high FT in the subset of patients with a hematologic malignancy (HR 2.39, 95% CI 1.17–4.85, *p* = 0.02). To address potential guarantee-time bias, an additional sensitivity analysis was done to determine survival from time of COST-FACIT completion (rather than from diagnosis) (Table 4). Moderate/high FT was associated with worse overall survival (HR 1.44, 95% CI 1.06–1.96, *p* = 0.02); which was consistent among patients with hematologic malignancies.

**Table 3:** Multivariable hazard ratios for overall survival following diagnosis by financial toxicity groups and ADI

Comparison	Events/*N*	Hazard ratio	Lower limit	Upper limit	*p*-value
*All patients*					
ADI (per 10%)	321/1623	1.03	0.99	1.08	0.17
Mod/high vs no impact	79/387 vs 130/693	1.16	0.85	1.57	0.34
Mod/high vs mild impact	79/387 vs 112/543	1.02	0.76	1.38	0.87
Mild vs no impact	112/543 vs 130/693	1.13	0.87	1.48	0.37
*Hematologic malignancy*					
ADI (per 10%)	72/498	0.97	0.88	1.07	0.57
** Mod/high vs no impact**	**21/128 vs 21/194**	**2.39**	**1.17**	**4.85**	***0.02***
Mod/high vs mild impact	21/128 vs 30/176	1.41	0.75	2.67	0.29
Mild vs no impact	30/176 vs 21/194	1.69	0.94	3.04	0.08
*Solid tumors*					
ADI (per 10%)	249/1125	1.04	0.99	1.09	0.11
Mod/high vs no impact	58/259 vs 109/499	1.03	0.72	1.49	0.86
Mod/high vs mild impact	58/259 vs 82/367	1.06	0.74	1.52	0.73
Mild vs no impact	82/367 vs 109/499	0.97	0.70	1.34	0.86

*N* = 1623 (all), *N* = 1125 (solid), and *N* = 498 (Hema). AUC = 0.73 (All), AUC = 0.72 (Solid), AUC = 0.75 (Hema). Adjusted for age, BMI, sex, race/ethnicity, primary payer, marital status, smoking status, clinical stage, and chemotherapy treatment

**Table 4:** Multivariable hazard ratios for overall survival following COST-FACIT completion by financial toxicity groups and ADI

Comparison	Hazard ratio	Lower limit	Upper limit	*p*-value
*All patients*				
ADI (per 10%)	1.03	0.99	1.08	0.19
** Mod/high vs no impact**	**1.44**	**1.06**	**1.96**	**0.02**
Mod/high vs mild impact	1.25	0.93	1.68	0.14
Mild vs no impact	1.16	0.89	1.51	0.28
*Hematologic malignancy*				
ADI (per 10%)	0.97	0.87	1.08	0.55
*** Mod/high vs no impact***	***4.02***	***1.94***	***8.36***	** < *0.001***
*** Mod/high vs mild impact***	***2.35***	***1.27***	***4.36***	***0.007***
Mild vs no impact	1.71	0.95	3.08	0.07
*Solid tumors*				
ADI (per 10%)	1.04	0.99	1.10	0.10
Mod/high vs no impact	1.19	0.83	1.70	0.36
Mod/high vs mild impact	1.16	0.81	1.66	0.43
Mild vs no impact	1.02	0.75	1.40	0.89

*N* = 1600 (All), *N* = 1110 (Solid), and *N* = 490 (Hema). AUC = 0.68 (All), AUC = 0.70 (Solid), AUC = 0.69 (Hema). Adjusted for age, BMI, sex, race/ethnicity, primary payer, marital status, smoking status, clinical stage, and chemotherapy treatment

There were also no significant differences in time-to-first treatment among patients reporting no, mild, and moderate/high FT. However, patients with higher ADI (per 10%) had longer time to treat first treatment (HR 0.98, 95% CI 0.96–1.00, *p* = 0.04) (Supplementary Table 3)

## Discussion

Our study describes financial toxicity as measured by COST-FACIT in a large real-world cohort of cancer patients seen at a tertiary care center with a FN program. We found several associations with higher FT including younger age, minority race and ethnicity, single marital status, Medicaid/Medicare insurance, smoking status, higher ADI, as well as higher distress and lower physical/mental health, all consistent with prior literature [23–26].

Although we did not initially find an association of higher FT with survival, an additional sensitivity analysis analyzing survival from time of COST-FACIT completion did demonstrate a significant association with moderate-high FT and worse survival. The median interval between diagnosis and COST-FACIT completion was 88 days, with 25% of assessments completed within 46 days and 10% completed within 28 days of diagnosis. This timing reflects the nature of financial toxicity as a patient-reported outcome that evolves after diagnosis and initiation of cancer care, rather than a baseline characteristic measurable at diagnosis. Nevertheless, we acknowledge that requiring survival until COST-FACIT completion introduces the possibility of guarantee-time bias. Additional clinical limitations may have influenced these findings, including heterogeneity of disease, treatment and patient characteristics of the cohort, and missing data.

Our current FN program at the Cleveland Clinic identifies all patients who are undergoing first treatment at the cancer center. After patients are scheduled for treatment, financial navigators review their benefits including insurance status, deductible, and maximum out-of-pocket expense. Patients are informed of their individualized costs with each treatment. Grant opportunities and medication programs and respective eligibility are evaluated based on diagnosis and medication. Barriers to care are also discussed. While the goal for the program is that the COST-FACIT is offered every 6 months, there remains missing longitudinal data, and current work is ongoing to optimize collection of longitudinal COST data to determine change in FT over time and potential impact of FN resources. The current data set is further limited by the lack of COST-FACIT data prior to the establishment of the FN program to better determine the impact of the FN program.

Although our cancer center is geographically positioned near multiple neighborhoods associated with high ADI, we found that patients with high ADI did have longer time to first treatment. The HR for this was small (2% longer time to first treatment) but still reflects a disparity. Our cancer center does track time to treat and has committed effort to improve this metric over time [27]. We also found that in the subset of patients with hematologic malignancies, moderate/high FT was associated with survival outcomes, while this was not found in solid tumor malignancies. Patients with hematologic malignancies are often initially diagnosed and initiate first treatment in the inpatient hospital setting and thus may not have the same initial FN touchpoint as those diagnosed with solid tumor malignancies; however this finding is exploratory and needs further investigation and validation.

In order to better understand if patients with a COST-FACIT survey were representative of our cancer center population, we performed sensitivity analyses which did highlight several differences between those who had a COST-FACIT survey and those who did not. Results thus may only be applicable to the described COST-FACIT population: patients were more likely to be younger, but also more likely to complete other PROMs (less missing data), and report higher distress by the NCCN DT. Non-Hispanic White patients were also more likely to complete the COST-FACIT compared to racial and ethnic minorities. While the reasons for non-completion are unfortunately not known for the entire cohort, anecdotal reports describe that many patients are reluctant to share financial information. As well, given the volume of patients seen across multiple sites and capacity of the financial navigators, patients may just not have received a COST survey. Thus, it is important to acknowledge the potential bias of patients who completed a COST and accordingly limits the generalizability of findings. In addition, while we acknowledge that this cohort was predominantly White, and the interpretation and significance of findings related to ethnic groups are limited by small numbers, we do note that among those who completed a COST-FACIT survey, approximately 18% were from minority groups; and these subgroups typically represent about 11% of the cancer center population.

We acknowledge several other limitations to this study, primarily due to its inclusion of a broad and heterogeneous cohort of patients. Several diseases were more representative than others, specifically breast, GI, leukemia and myeloma; whereas diagnosis of genitourinary, gynecological, head and neck, and skin cancers was under-represented; and thus, this study may lack generalizability to specific diagnoses. We also acknowledge and know from existing data that FT, time-to-treat, and survival outcomes may vary based on comorbidity, disease, stage, and treatment approach; however, the purpose of this study was to describe and provide a scoping overview of the burden of FT at a large cancer center. These initial results may inform future studies within specific subgroups, particularly as we continue to gather longitudinal data. As noted previously, optimization of follow-up COST-FACIT data will better inform the impact of a FN program and FN interventions. In addition, while we hope to better understand the relationships and interplay between health-related quality of life (HRQOL) and FT, based on the timing of routine PROM data collection and COST-FACIT results, it is not possible to infer causality or directionality of FT and other PROMs. As longitudinal data is collected, we plan to analyze the dynamic aspects of HRQOL and FT and how it may fluctuate with the clinical course.

In summary, this analysis confirms a significant burden of FT in our cancer population, as well as several factors associated with FT. We did not find any association of higher FT and survival or time-to-treat outcome. Further study is needed to better understand these findings within specific disease groups and the longitudinal impact of financial navigation on FT.

## Supplementary Information

Below is the link to the electronic supplementary material.

ESM 1 DOCX (77.9 KB)

## Data Availability

All data supporting the findings of this study are available within the paper and its Supplementary Information.

## References

[1] Lentz R, Benson AB 3rd, Kircher S (2019) Financial toxicity in cancer care: prevalence, causes, consequences, and reduction strategies. J Surg Oncol 120(1):85–92 10.1002/jso.25374 30650186

[2] Mariotto AB, Enewold L, Zhao J et al (2020) Medical care costs associated with cancer survivorship in the United States. Cancer Epidemiol Biomarkers Prev 29:1304–1312 10.1158/1055-9965.EPI-19-1534 PMC9514601 32522832

[3] de Boer AG, Taskila T, Ojajarvi A et al (2009) Cancer survivors and unemployment: a meta-analysis and meta-regression. JAMA 301:753–762 10.1001/jama.2009.187 19224752

[4] Dowling EC, Chawla N, Forsythe LP et al (2013) Lost productivity and burden of illness in cancer survivors with and without other chronic conditions. Cancer 119:3393–3401 10.1002/cncr.28214 PMC5861717 23794146

[5] Guy GP Jr, Ekwueme DU, Yabroff KR et al (2013) Economic burden of cancer survivorship among adults in the United States. J Clin Oncol 31:3749–3757 10.1200/JCO.2013.49.1241 PMC3795887 24043731

[6] Lathan CS, Cronin A, Tucker-Seeley R et al (2016) Association of financial strain with symptom burden and quality of life for patients with lung or colorectal cancer. J Clin Oncol 34:1732–1740 10.1200/JCO.2015.63.2232 PMC4966336 26926678

[7] Ramsey SD, Bansal A, Fedorenko CR et al (2016) Financial insolvency as a risk factor for early mortality among patients with cancer. J Clin Oncol 34:980–986 10.1200/JCO.2015.64.6620 PMC4933128 26811521

[8] Pangestu S, Rencz F (2023) Comprehensive score for financial toxicity and health-related quality of life in patients with cancer and survivors: a systematic review and meta-analysis. Value Health 26:300–316 10.1016/j.jval.2022.07.017 36064514

[9] Zafar SY, Peppercorn JM, Schrag D et al (2013) The financial toxicity of cancer treatment: a pilot study assessing out-of-pocket expenses and the insured cancer patient’s experience. Oncologist 18:381–90 10.1634/theoncologist.2012-0279 PMC3639525 23442307

[10] Doshi JA, Li P, Huo H et al (2018) Association of patient out-of-pocket costs with prescription abandonment and delay in fills of novel oral anticancer agents. J Clin Oncol 36:476–482 10.1200/JCO.2017.74.5091 29261440

[11] Kent EE, Forsythe LP, Yabroff KR et al (2013) Are survivors who report cancer-related financial problems more likely to forgo or delay medical care? Cancer 119:3710–3717 10.1002/cncr.28262 PMC4552354 23907958

[12] Zullig LL, Peppercorn JM, Schrag D et al (2013) Financial distress, use of cost-coping strategies, and adherence to prescription medication among patients with cancer. J Oncol Pract 9:60s–63s 10.1200/JOP.2013.000971 PMC3825170 29431038

[13] Yabroff KR, Han X, Song W et al (2022) Association of medical financial hardship and mortality among cancer survivors in the United States. J Natl Cancer Inst 114:863–870 10.1093/jnci/djac044 PMC9194618 35442439

[14] Dee EC, Nipp RD, Muralidhar V et al (2013) Financial worry and psychological distress among cancer survivors in the United States, 2013-2018. Support Care Cancer 29:5523–5535 10.1007/s00520-021-06084-1 33725174

[15] Kale HP, Carroll NV (2016) Self-reported financial burden of cancer care and its effect on physical and mental health-related quality of life among US cancer survivors. Cancer 122:283–289 10.1002/cncr.29808 26991528

[16] Ver Hoeve ES, Ali-Akbarian L, Price SN et al (2021) Patient-reported financial toxicity, quality of life, and health behaviors in insured US cancer survivors. Support Care Cancer 29:349–358 10.1007/s00520-020-05468-z PMC9208736 32361832

[17] Wheeler SB, Manning ML, Gellin M et al (2024) Impact of a comprehensive financial navigation intervention to reduce cancer-related financial toxicity. J Natl Compr Canc Netw 22:557–562 10.6004/jnccn.2024.7030 PMC12690979 39413831

[18] de Souza JA, Yap BJ, Wroblewski K et al (2017) Measuring financial toxicity as a clinically relevant patient-reported outcome: the validation of the COmprehensive score for financial Toxicity (COST). Cancer 123:476–484 10.1002/cncr.30369 PMC5298039 27716900

[19] De Souza JA, Wroblewski K, Proussaloglou E et al (2017) Validation of a financial toxicity (FT) grading system. J Clin Oncol. 10.1200/JCO.2017.35.15_suppl.6615

[20] Kind AJH, Buckingham WR (2018) Making neighborhood-disadvantage metrics accessible - the Neighborhood Atlas. N Engl J Med 378:2456–2458 10.1056/NEJMp1802313 PMC6051533 29949490

[21] University of Wisconsin School of Medicine and Public Health. 2013–2017 Area Deprivation Index v2.0. Downloaded from https://www.neighborhoodatlas.medicine.wisc.edu/ Dec 14, 2025,

[22] Hosmer DW, Lemeshow S (2000) Applied logistic regression. Wiley, New York

[23] Kadambi S, Wang Y, Job A et al (2025) Financial toxicity in older adults with cancer and their caregivers. JCO Oncol Pract 21:92–99 10.1200/OP.24.00105 PMC11731888 39793550

[24] Benedict C, Fisher S, Schapira L et al (2022) Greater financial toxicity relates to greater distress and worse quality of life among breast and gynecologic cancer survivors. Psychooncology 31:9–20 10.1002/pon.5763 PMC9809212 34224603

[25] Syed N, Dusetzina S, Koyama T et al (2024) Insurance, financial strain, family function, and psychosocial wellbeing in pediatric patients and parents with hematologic malignancies. Blood 144:2279–2279

[26] Hsu ML, Boulanger MC, Olson S et al (2024) Unmet needs, quality of life, and financial toxicity among survivors of lung cancer. JAMA Netw Open 7:e246872 10.1001/jamanetworkopen.2024.6872 PMC11024770 38630475

[27] Bolwell BJ, Khorana AA (2016) Enhancing value for patients with cancer: time to treatment as a surrogate for integrated cancer care. J Natl Compr Canc Netw 14:115–6 10.6004/jnccn.2016.0010 26733558

