# Supplementary material for: Assessing financial toxicity with COST-FACIT: experience from a tertiary care cancer center
Source: Support Care Cancer. 2026 Oct 17;34(11):1119. doi: 10.1007/s00520-026-11344-z (PMC13676559; doi:10.1007/s00520-026-11344-z)
Supplement: Supplementary file 1 [file 520_2026_11344_MOESM1_ESM.docx]

**Supplementary Figure 1:**

Supplementary Table 1: Association of Diagnosis and Treatments with Financial Toxicity Group

| **Factor** | **Total (N=1,906)** | **No impact (N=815)** | **Mild impact (N=632)** | **Moderate impact (N=414)** | **High impact (N=45)** | **p-value** |  |
| --- | --- | --- | --- | --- | --- | --- | --- |
| Diagnosis |  |  |  |  |  | 0.31^c^ |  |
| Breast | 305 (16.0) | 122 (15.0) | 99 (15.7) | 73 (17.6) | 11 (24.4) |  |  |
| GI | 347 (18.2) | 159 (19.5) | 115 (18.2) | 66 (15.9) | 7 (15.6) |  |  |
| GU | 183 (9.6) | 93 (11.4) | 60 (9.5) | 29 (7.0) | 1 (2.2) |  |  |
| Gyn | 88 (4.6) | 43 (5.3) | 28 (4.4) | 17 (4.1) | 0 (0.00) |  |  |
| Head and Neck | 93 (4.9) | 37 (4.5) | 30 (4.7) | 23 (5.6) | 3 (6.7) |  |  |
| Leukemia, MDS | 135 (7.1) | 44 (5.4) | 54 (8.5) | 36 (8.7) | 1 (2.2) |  |  |
| Lung | 230 (12.1) | 104 (12.8) | 71 (11.2) | 50 (12.1) | 5 (11.1) |  |  |
| Lymphoma | 245 (12.9) | 98 (12.0) | 77 (12.2) | 60 (14.5) | 10 (22.2) |  |  |
| Myeloma | 196 (10.3) | 78 (9.6) | 69 (10.9) | 43 (10.4) | 6 (13.3) |  |  |
| Other | 34 (1.8) | 15 (1.8) | 12 (1.9) | 7 (1.7) | 0 (0.00) |  |  |
| Skin inc Melanoma, Merkel | 50 (2.6) | 22 (2.7) | 17 (2.7) | 10 (2.4) | 1 (2.2) |  |  |
| Clinical Stage |  |  |  |  |  | 0.97^c^ |  |
| 0 | 31 (1.6) | 14 (1.7) | 11 (1.7) | 5 (1.2) | 1 (2.2) |  |  |
| I | 280 (14.7) | 121 (14.8) | 94 (14.9) | 59 (14.3) | 6 (13.3) |  |  |
| II | 206 (10.8) | 90 (11.0) | 65 (10.3) | 48 (11.6) | 3 (6.7) |  |  |
| III | 197 (10.3) | 84 (10.3) | 69 (10.9) | 41 (9.9) | 3 (6.7) |  |  |
| IV | 290 (15.2) | 128 (15.7) | 85 (13.4) | 67 (16.2) | 10 (22.2) |  |  |
| Unknown | 902 (47.3) | 378 (46.4) | 308 (48.7) | 194 (46.9) | 22 (48.9) |  |  |
| Treatment: chemotherapy | 962 (50.5) | 398 (48.8) | 323 (51.1) | 221 (53.4) | 20 (44.4) | 0.39^c^ |  |
| Chemotherapy Treatments |  |  |  |  |  | 0.83^c^ |  |
| Multiple Agents | 679 (35.6) | 279 (34.2) | 233 (36.9) | 152 (36.7) | 15 (33.3) |  |  |
| No Chemotherapy | 953 (50.0) | 420 (51.5) | 310 (49.1) | 198 (47.8) | 25 (55.6) |  |  |
| Single Agent | 274 (14.4) | 116 (14.2) | 89 (14.1) | 64 (15.5) | 5 (11.1) |  |  |
| Treatment: radiation | 472 (24.8) | 201 (24.7) | 156 (24.7) | 109 (26.3) | 6 (13.3) | 0.29^c^ |  |
| Treatment: immunotherapy | 492 (25.8) | 199 (24.4) | 166 (26.3) | 115 (27.8) | 12 (26.7) | 0.63^c^ |  |
| Treatment: hormone therapy | 340 (17.8) | 126 (15.5) | 130 (20.6) | 76 (18.4) | 8 (17.8) | 0.092^c^ |  |
| Treatment: other | 44 (2.3) | 19 (2.3) | 16 (2.5) | 8 (1.9) | 1 (2.2) | 0.94^c^ |  |
| Treatment: surgery | 580 (30.4) | 250 (30.7) | 197 (31.2) | 123 (29.7) | 10 (22.2) | 0.63^c^ |  |
| Treatment: Chemotherapy + Radiation | 343 (18.0) | 137 (16.8) | 112 (17.7) | 89 (21.5) | 5 (11.1) | 0.13^c^ |  |
| Treatment: Chemotherapy + Immunotherapy | 420 (22.0) | 168 (20.6) | 139 (22.0) | 104 (25.1) | 9 (20.0) | 0.34^c^ |  |
| Treatment: Chemotherapy + Hormone therapy | 276 (14.5) | 97 (11.9) | 110 (17.4) | 62 (15.0) | 7 (15.6) | ***0.031^c^*** |  |
| Treatment: Immunotherapy + Radiation | 150 (7.9) | 59 (7.2) | 47 (7.4) | 42 (10.1) | 2 (4.4) | 0.23^c^ |  |
| Treatment: Radiation + Hormone therapy | 121 (6.3) | 50 (6.1) | 41 (6.5) | 30 (7.2) | 0 (0.00) | 0.30^c^ |  |
| Treatment: Immunotherapy + Hormone therapy | 189 (9.9) | 67 (8.2) | 71 (11.2) | 47 (11.4) | 4 (8.9) | 0.18^c^ |  |
| Number of Treatments |  |  |  |  |  | 0.64^c^ |  |
| 0 | 723 (37.9) | 314 (38.5) | 232 (36.7) | 157 (37.9) | 20 (44.4) |  |  |
| 1 | 449 (23.6) | 210 (25.8) | 147 (23.3) | 82 (19.8) | 10 (22.2) |  |  |
| 2 | 402 (21.1) | 163 (20.0) | 136 (21.5) | 94 (22.7) | 9 (20.0) |  |  |
| 3 | 272 (14.3) | 106 (13.0) | 96 (15.2) | 65 (15.7) | 5 (11.1) |  |  |
| 4 | 59 (3.1) | 21 (2.6) | 21 (3.3) | 16 (3.9) | 1 (2.2) |  |  |
| 5 | 1 (0.05) | 1 (0.12) | 0 (0.00) | 0 (0.00) | 0 (0.00) |  |  |

Statistics presented as N (column %). p-values: c=Pearson's chi-square test.

Supplementary Table 2 Sensitivity Analysis between patients with filled out COST FACIT and who did not

|  | **CF Cohort** | | **NO CF** | | | **No CF, No PROMs** | | | **No CF, but 1+ PROM** | | |
| --- | --- | --- | --- | --- | --- | --- | --- | --- | --- | --- | --- |
|  | **(N=1,906)** | | **(N=14,517)** | | | **(N=3,020)** | | | **(N=11,497)** | | |
| **Factor** | **N** | **Statistics** | **N** | **Statistics** | **p-value** | **N** | **Statistics** | **p-value** | **N** | **Statistics** | **p-value** |
| Age | 1,906 | 65.0 [56.0, 72.0] | 14,517 | 69.0 [60.0, 77.0] | ***<0.001^b^*** | 3,020 | 71.0 [62.0, 79.0] | ***<0.001^b^*** | 11,497 | 68.0 [59.0, 76.0] | ***<0.001^b^*** |
| BMI | 1,905 | 26.9 [23.4, 30.5] | 14,517 | 27.4 [23.7, 31.8] | ***0.031^b^*** | 3,020 | 26.7 [23.1, 30.9] | ***0.019^b^*** | 11,497 | 27.6 [23.9, 32.1] | ***<0.001^b^*** |
| NCCN Score | 1,001 | 3.0 [0.00, 5.0] | 9,681 | 2.0 [0.00, 5.0] | ***0.025^b^*** | 0 | --- |  | 9,681 | 2.0 [0.00, 5.0] | ***0.025^b^*** |
| PROMIS-10 MENTAL | 1,474 | 45.8 [41.1, 53.3] | 10,612 | 45.8 [41.1, 53.3] | 0.091^b^ | 0 | --- |  | 10,612 | 45.8 [41.1, 53.3] | 0.091^b^ |
| PROMIS-10 PHYSICAL | 1,505 | 44.9 [37.4, 50.8] | 10,678 | 44.9 [39.8, 50.8] | 0.24^b^ | 0 | --- |  | 10,678 | 44.9 [39.8, 50.8] | 0.24^b^ |
| PHQ9 Score | 0 | --- | 4,011 | 7.0 [3.0, 12.0] |  | 0 | --- |  | 4,011 | 7.0 [3.0, 12.0] |  |
| Missing Promise-10 Physical | 1,906 | 401 (21.0) | 14,517 | 3,839 (26.4) | ***<0.001^c^*** | 3,020 | 3,020 (100.0) | ***<0.001^c^*** | 11,497 | 819 (7.1) | ***<0.001^c^*** |
| Missing Promise-10 Mental | 1,906 | 432 (22.7) | 14,517 | 3,905 (26.9) | ***<0.001^c^*** | 3,020 | 3,020 (100.0) | ***<0.001^c^*** | 11,497 | 885 (7.7) | ***<0.001^c^*** |
| Missing NCCN | 1,906 | 905 (47.5) | 14,517 | 4,836 (33.3) | ***<0.001^c^*** | 3,020 | 3,020 (100.0) | ***<0.001^c^*** | 11,497 | 1,816 (15.8) | ***<0.001^c^*** |
| Missing PHQ-2 | 1,906 | 490 (25.7) | 14,517 | 4,118 (28.4) | ***0.015^c^*** | 3,020 | 3,020 (100.0) | ***<0.001^c^*** | 11,497 | 1,098 (9.6) | ***<0.001^c^*** |
| Missing PHQ-9 | 1,906 | 1,828 (95.9) | 14,517 | 10,245 (70.6) | ***<0.001^c^*** | 3,020 | 3,017 (99.9) | ***<0.001^c^*** | 11,497 | 7,228 (62.9) | ***<0.001^c^*** |
| PHQ 2 Score | 1,416 |  | 10,399 |  | ***<0.001^c^*** | 0 |  |  | 10,399 |  | ***<0.001^c^*** |
| 0 |  | 849 (60.0) |  | 6,380 (61.4) |  |  | --- |  |  | 6,380 (61.4) |  |
| 1 |  | 204 (14.4) |  | 1,291 (12.4) |  |  | --- |  |  | 1,291 (12.4) |  |
| 2 |  | 271 (19.1) |  | 1,628 (15.7) |  |  | --- |  |  | 1,628 (15.7) |  |
| 3 |  | 32 (2.3) |  | 404 (3.9) |  |  | --- |  |  | 404 (3.9) |  |
| 4 |  | 31 (2.2) |  | 375 (3.6) |  |  | --- |  |  | 375 (3.6) |  |
| 5 |  | 7 (0.49) |  | 128 (1.2) |  |  | --- |  |  | 128 (1.2) |  |
| 6 |  | 22 (1.6) |  | 193 (1.9) |  |  | --- |  |  | 193 (1.9) |  |
| Race | 1,906 |  | 14,517 |  | ***<0.001^c^*** | 3,020 |  | ***<0.001^c^*** | 11,497 |  | ***<0.001^c^*** |
| Non-Hispanic White |  | 1,561 (81.9) |  | 11,346 (78.2) |  |  | 2,096 (69.4) |  |  | 9,250 (80.5) |  |
| Non-Hispanic Black |  | 237 (12.4) |  | 1,812 (12.5) |  |  | 503 (16.7) |  |  | 1,309 (11.4) |  |
| Hispanic |  | 45 (2.4) |  | 784 (5.4) |  |  | 266 (8.8) |  |  | 518 (4.5) |  |
| Unknown/Declined |  | 63 (3.3) |  | 575 (4.0) |  |  | 155 (5.1) |  |  | 420 (3.7) |  |
| Marital Status | 1,885 |  | 14,388 |  | 0.060^c^ | 2,978 |  | ***0.010^c^*** | 11,410 |  | ***0.003^c^*** |
| Single |  | 406 (21.5) |  | 2,768 (19.2) |  |  | 682 (22.9) |  |  | 2,086 (18.3) |  |
| Married |  | 1,091 (57.9) |  | 8,562 (59.5) |  |  | 1,596 (53.6) |  |  | 6,966 (61.1) |  |
| Divorced/Separated/Widowed |  | 388 (20.6) |  | 3,058 (21.3) |  |  | 700 (23.5) |  |  | 2,358 (20.7) |  |
| Primary Payer | 1,906 |  | 14,517 |  | ***<0.001^c^*** | 3,020 |  | ***<0.001^c^*** | 11,497 |  | ***<0.001^c^*** |
| Medicare |  | 665 (34.9) |  | 1,873 (12.9) |  |  | 353 (11.7) |  |  | 1,520 (13.2) |  |
| Medicaid |  | 211 (11.1) |  | 1,551 (10.7) |  |  | 386 (12.8) |  |  | 1,165 (10.1) |  |
| Private |  | 896 (47.0) |  | 10,318 (71.1) |  |  | 2,117 (70.1) |  |  | 8,201 (71.3) |  |
| Other |  | 134 (7.0) |  | 775 (5.3) |  |  | 164 (5.4) |  |  | 611 (5.3) |  |
| Sex | 1,905 |  | 14,517 |  | 0.71^c^ | 3,020 |  | ***0.016^c^*** | 11,497 |  | 0.77^c^ |
| FEMALE |  | 1,033 (54.2) |  | 7,807 (53.8) |  |  | 1,531 (50.7) |  |  | 6,276 (54.6) |  |
| MALE |  | 872 (45.8) |  | 6,710 (46.2) |  |  | 1,489 (49.3) |  |  | 5,221 (45.4) |  |
| Diagnosis | 1,872 |  | 14,205 |  | ***<0.001^c^*** | 2,978 |  | ***<0.001^c^*** | 11,227 |  | ***<0.001^c^*** |
| Breast |  | 305 (16.3) |  | 2,478 (17.4) |  |  | 359 (12.1) |  |  | 2,119 (18.9) |  |
| GI |  | 347 (18.5) |  | 2,990 (21.0) |  |  | 677 (22.7) |  |  | 2,313 (20.6) |  |
| GU |  | 183 (9.8) |  | 1,666 (11.7) |  |  | 399 (13.4) |  |  | 1,267 (11.3) |  |
| Gyn |  | 88 (4.7) |  | 692 (4.9) |  |  | 161 (5.4) |  |  | 531 (4.7) |  |
| Head and Neck |  | 93 (5.0) |  | 637 (4.5) |  |  | 114 (3.8) |  |  | 523 (4.7) |  |
| Ill defined Malignancy |  | 0 (0.00) |  | 288 (2.0) |  |  | 78 (2.6) |  |  | 210 (1.9) |  |
| Leukemia, MDS |  | 135 (7.2) |  | 1,036 (7.3) |  |  | 242 (8.1) |  |  | 794 (7.1) |  |
| Lung |  | 230 (12.3) |  | 1,834 (12.9) |  |  | 388 (13.0) |  |  | 1,446 (12.9) |  |
| Lymphoma |  | 245 (13.1) |  | 1,252 (8.8) |  |  | 229 (7.7) |  |  | 1,023 (9.1) |  |
| Myeloma |  | 196 (10.5) |  | 1,012 (7.1) |  |  | 239 (8.0) |  |  | 773 (6.9) |  |
| Skin including  Melanoma, Merkel |  | 50 (2.7) |  | 320 (2.3) |  |  | 92 (3.1) |  |  | 228 (2.0) |  |
| Type of Tumor | 1,906 |  | 14,517 |  | ***<0.001^c^*** | 3,020 |  | ***<0.001^c^*** | 11,497 |  | ***<0.001^c^*** |
| Hemangioma |  | 576 (30.2) |  | 3,300 (22.7) |  |  | 710 (23.5) |  |  | 2,590 (22.5) |  |
| Solid |  | 1,330 (69.8) |  | 11,217 (77.3) |  |  | 2,310 (76.5) |  |  | 8,907 (77.5) |  |

Statistics presented as Median [P25, P75], N (column %).

p-values: b=Wilcoxon Rank Sum test, c=Pearson's chi-square test.

Supplementary Table 3 Multivariable hazard rations for Time to First Treatment

| **Comparison** | **Hazard Ratio** | **Lower Limit** | **Upper Limit** | **p-value** |
| --- | --- | --- | --- | --- |
| *All Patients* |  |  |  |  |
| ***ADI (per 10%)*** | ***0.98*** | ***0.96*** | ***1.00*** | ***0.04*** |
| Mod/High vs No Impact | 1.02 | 0.89 | 1.16 | 0.82 |
| Mod/High vs Mild Impact | 0.94 | 0.82 | 1.08 | 0.37 |
| Mild vs No Impact | 1.08 | 0.96 | 1.21 | 0.19 |
| *Hematologic malignancy* |  |  |  |  |
| ADI (per 10%) | 0.99 | 0.96 | 1.02 | 0.53 |
| Mod/High vs No Impact | 0.91 | 0.73 | 1.15 | 0.44 |
| Mod/High vs Mild Impact | 0.82 | 0.64 | 1.03 | 0.09 |
| Mild vs No Impact | 1.12 | 0.89 | 1.41 | 0.34 |
| *Solid Tumors* |  |  |  |  |
| ADI (per 10%) | 0.98 | 0.96 | 1.00 | 0.12 |
| Mod/High vs No Impact | 1.11 | 0.94 | 1.31 | 0.23 |
| Mod/High vs Mild Impact | 1.00 | 0.84 | 1.18 | 0.96 |
| Mild vs No Impact | 1.11 | 0.98 | 1.27 | 0.11 |

N=1,623 (All), N=1,125 (Solid), N=498 (Hema). AUC=0.64 (All), AUC=0.64 (Solid) and

AUC=0.69 (Hema). Adjusted for age, BMI, sex, race/ethnicity, primary payer, marital status,

smoking status, clinical stage, and chemotherapy treatment.
